# Etiologic Classification of 541 Infantile Spasms Cases: A Cohort Study

**DOI:** 10.3389/fped.2022.774828

**Published:** 2022-03-07

**Authors:** Pan Peng, Miriam Kessi, Leilei Mao, Fang He, Ciliu Zhang, Chen Chen, Nan Pang, Fei Yin, Zou Pan, Jing Peng

**Affiliations:** ^1^Department of Pediatrics, Xiangya Hospital, Central South University, Changsha, China; ^2^Hunan Intellectual and Developmental Disabilities Research Center, Changsha, China

**Keywords:** infantile spasms (IS), etiologies, spectrum, variants, whole-exome sequencing (WES)

## Abstract

**Objective:**

To explore the etiology of infantile spasms (IS) in a large Chinese cohort based on the United States National Infantile Spasms Consortium (NISC) classification.

**Methods:**

In the present study, we recruited IS patients diagnosed at a single center (Xiangya Hospital, Central South University) between Jan 2010 and Aug 2019. Thereafter, we collected their clinical and genetic information retrospectively. Their underlying etiologies were classified according to the NISC classification and then compared in different scenarios to understand their distribution.

**Results:**

A total of 541 patients with IS from 18 provinces were included in this study. The underlying etiology was identified in 53.2% of the cases: structural-acquired, 25.3%; genetic, 12.9%; genetic-structural, 7.2%; structural-congenital, 5.0%; metabolic, 2.4%; infections, 0.4% and immune, 0%. Whole-exome sequencing (WES) provided the highest diagnostic yield (26.9%). In structural-acquired IS, the proportion of hypoglycemic brain injuries was significant, second only to hypoxic-ischemic encephalopathy. There was no patient discovered to have Down syndrome. *STXBP1, CDKL5, TSC2, KCNQ2, IRF2BPL*, and *TSC1* were the most frequently implicated genes. Genetic causes were found to be the most common cause of IS in the early onset group, while structural-acquired etiologies were common in males and preterm babies. Patients with pre-spasm seizures were associated with a higher proportion of identified causes than those without. Non-acquired structural etiologies were more common in patients without hypsarrhythmia than in those with hypsarrhythmia.

**Significance:**

The most prevalent cause of IS was structural acquired followed by genetic causes. When brain MRI fails to detect the etiology, we propose WES as the next step. Structural-acquired IS and cases with genetic disorders are characteristic of the Chinese cohort, however, the etiology differs with the patient's age of onset, gestation age at birth, sex, and the presence/absence of both pre-spasm seizures, and hypsarrhythmia.

## Highlights

- More than half of the IS cases in China have an underlying etiology.
- The most common cause is structural acquired followed by genetic.
- Both males and preterm babies independently have higher proportions of structural-acquired etiologies than their counterparts.
- Early-onset IS patients have a larger proportion of genetic reasons than other onset ages, with *STXBP1* being the most prevalent causative gene.

## Introduction

Infantile spasms (IS) is a type of developmental and epileptic encephalopathy (DEE), with an incidence of 0.43 per 1,000 live births, occurs mostly between the ages of 3 and 12 months, and with a peak occurring around 4–7 months (1–3). Studies have shown that nearly 60% of the cases have an underlying etiology (4–6), however, data from the Chinese population is lacking. Moreover, only a few studies have explored the distribution and etiology of IS in different scenarios (5, 7).

In 2015, the National Infantile Spasms Consortium (NISC) in North America divided the etiologies into eight groups, including genetic, genetic-structural, structural-congenital, structural-acquired, metabolic, immune, infectious, and unknown etiologies (8). Considering the rationality and operability of this consortium, we adopted it as the standard etiological classification and used it to investigate the underlying etiology of 541 cases of IS in the central south of China. Moreover, this study also explored the distribution of the patients based on different scenarios, such as the age of onset, gestation age at birth, sex, and the presence/absence of both pre-spasm seizures, and hypsarrhythmia to guide clinical management.

## Materials and Methods

### Study Participants

All individuals with IS seen at Xiangya Hospital, Central South University between Jan 2010 and Aug 2019 were retrospectively enrolled in this study. We included cases with: (1) epileptic spasms manifested within 2 years of age and (2) typical findings in electroencephalography (EEG), such as hypsarrhythmia or modified hypsarrhythmia. Cases without hypsarrhythmia, but with a background characterized by multifocal spikes and electroclinical spasms were also included.

### Diagnostic Protocol

**Figure 1:**
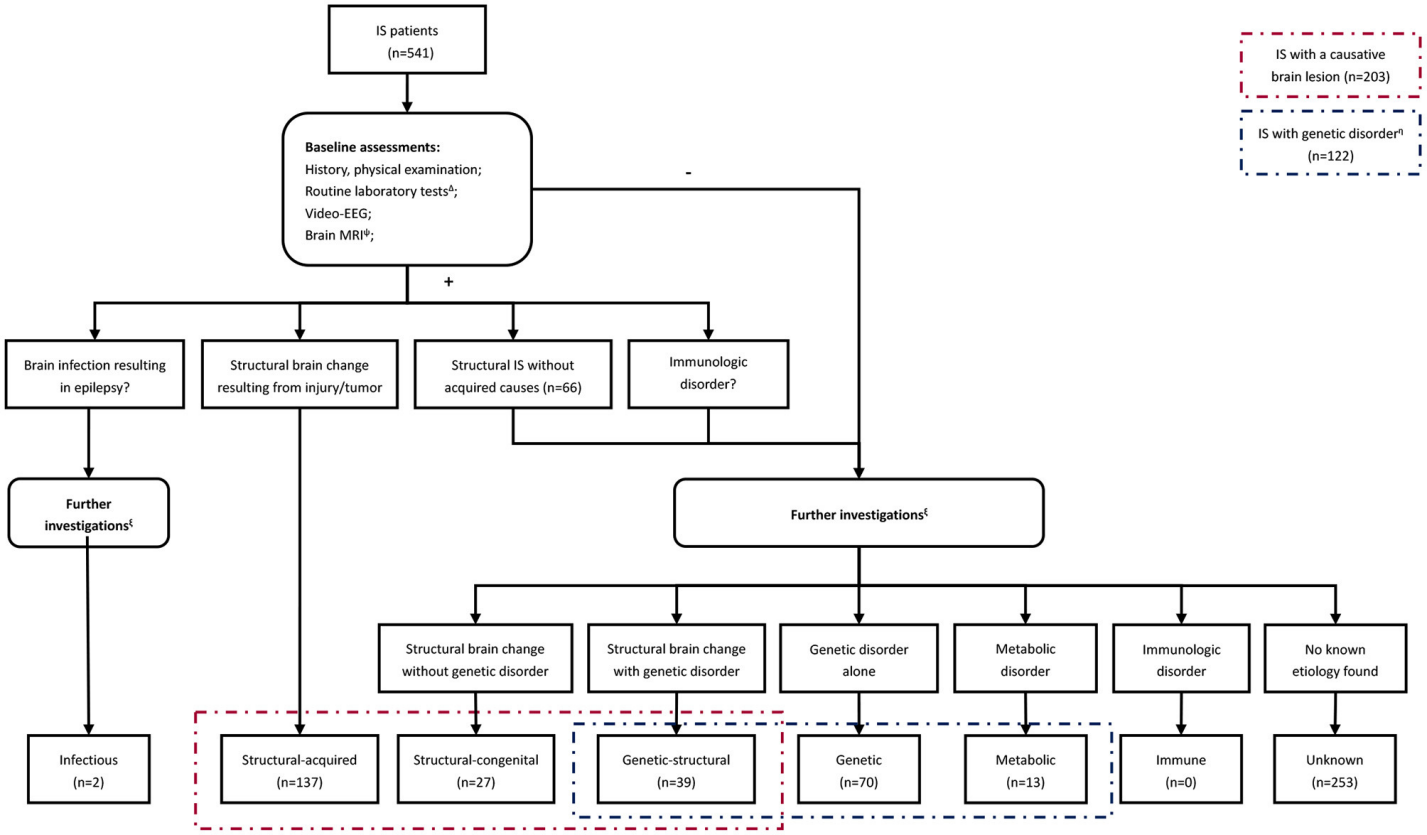
The flow chart summarizing the steps involved in identifying the underlying etiology. Δ Tier1 laboratory investigations included complete blood count, glucose, electrolytes, urea, creatinine, AST, ALT, creatine kinase, ammonia, lactate, homocysteine, and ceruloplasmin. Ψ Brain MRI was rechecked after several months or cranial MRI thin slice scan/positron emission tomography-computed tomography (PET-CT) was performed on patients who had focal clinical seizures or focal abnormalities on EEG. ξ Further investigations included infectious or immunological tests, plasma amino acids, urine organic acids, karyotype analysis, chromosomal microarray analysis, customized multigene panel, whole-exome sequencing, and mitochondrial genome analysis. The performance of these investigations was based on neurologists' judgments of the probable etiology and varied among the patients. Forty-six patients refused any further investigations in this study. η There were 103 cases with available genetic data among those with a genetic disorder, while the remaining 17 met the clinical diagnostic criteria for tuberous sclerosis complex and 2 for neurofibromatosis.

A stepwise approach was employed to identify the underlying etiology for each case based on the NISC classification (Figure 1). It's worth noting that individuals with acquired intracranial lesions resulting in epilepsy were classified into the structural-acquired group, including those caused by an intracranial infection in this study. Besides, identification of the probable etiology was dependent on the neurologists' judgment, which varied among the patients and period.

Patient follow-up: a repeat brain imaging was performed for patients who had focal clinical seizures or focal EGG abnormalities. For patients suspected to have genetic disorders, raw data of genetic tests were re-analyzed periodically. Recently published literature reviews and scientific studies on bioinformatics analysis, RNA-seq, *in vivo*, and *in vitro* experiments helped with the interpretation of sequencing data.

### Data Collection

The following data were retrieved during the patient's visit to the hospital: demographics, seizure-related information, relevant medical history, and medical record abstraction were performed. Individuals with insufficient medical records or lacked baseline assessments were excluded from the study. All data were independently reviewed by two neurologists. Furthermore, at least one neuro-radiologist reviewed the brain images, and all genetic results were interpreted by two geneticists following the American College of Medical Genetics (ACMG) guidelines (9, 10). A third geneticist was consulted to reach a consensus in case of disagreement. Patients with pathogenic/likely pathogenic copy number variations (CNVs) were included in the group with identified causes, while the others were categorized as unknown IS. Patients with pathogenic/likely pathogenic/uncertain significance variants were referred to neurologists for a literature review of genotype-phenotype correlation. Specific etiologies were recorded and classified into appropriate NISC categories based on the preceding work.

A systematic search of all the identified genes in the Online Mendelian Inheritance in Man (OMIM) and PubMed databases was performed. A thorough literature review was conducted to present the general perspective of all the causal genes, including the gene types, functions, and treatment conditions.

### Statistical Analysis

Statistical analysis was performed using the Statistical Package for Social Science (IBM, SPSS Statistics Version 25). Categorical data were summarized in the form of frequencies and proportions and analyzed with the Chi-square test (*p* ≤ 0.05 indicated statistically significant differences between groups) or Fisher's exact test with a Bonferroni adjustment (*p* ≤ 0.0167, *k* = 3) where applicable.

### Ethical Standards

This study was reviewed and approved by the Institutional Ethics Committee of Xiangya Hospital, Central South University, and it was performed following the World Medical Association Declaration of Helsinki adopted in 1964. Written informed consent was obtained from the parents/guardians of the subjects.

## Results

### The Demographic Information of the Cohort

A total of 541 patients with IS from 18 provinces were included as shown in Supplementary Figure S1. The patient's demographics were presented in Table 1.

**Table 1:** The baseline characteristics of the group.

**Variable**	**Total patients (*N*) =541**	**Percentage**
**Sex**		
Male	330	61.0%
Female	211	39.0%
**Ethnicity**		
Han	514	95.0%
Non-Han	27	5.0%
**Classification according to the age at spasms onset (corrected for preterm delivery)**		
Early-onset, <3 m	86	15.9%
Classic-onset, ≥3 to <12 m	393	72.6%
Late-onset, ≥12 m	62	11.5%
**Preceding seizures**		
Yes	133	24.6%
No	408	75.4%
**Unequivocally normal development at the onset of spasms**		
Yes	161	29.8%
No	380	70.2%
**Gestational age**		
<32 w	17	3.1%
≥32 to <37 w	33	6.1%
≥37 w	491	90.8%
**Birthweight**		
<1,500 g	10	1.9%
≥1,500 to <2,500 g	46	8.5%
≥2,500 to <4,000 g	461	85.2%
≥4,000 g	24	4.4%
**Presence of hypsarrhythmia in EEG**		
Yes	467	86.3%
No	74	13.7%
**Presence of spasms in clusters**		
Yes	518	95.7%
No	23	4.3%

*EEG, electroencephalograph; g, gram; m, month; w, week*.

### Diagnostic Yield

#### Neuroimaging

Brain magnetic resonance images (MRIs) were available for all 541 cases. The causative lesions were identified in 203 (37.5%) cases, of which 137 had structural-acquired lesions, while 66 had non-acquired structural lesions (genetic/congenital-structural). Eight (3.9%) patients with causal structural abnormalities were revealed by repeated brain MRI.

#### Genetic and Metabolic Tests

After completing baseline assessments, 402 cases without obvious acquired causes (structural-acquired and infectious IS) were transferred for additional genetic and/or metabolic testing. Of the 402 cases, 46 refused any further investigations while the remaining 356 cases underwent genetic and/or metabolic testing. Of the 305 cases who underwent genetic testing, 103 (33.8%) had an established genetic disorder. The diagnostic yield for each test was as follows: plasma amino acids and urine organic acids (1/309, 0.3%), karyotype (2/183, 1.1%), CMA (12/207, 5.8%), customized multigene panels (27/105, 25.7%), WES (63/234, 26.9%; nine of the 63 patients were found through customized multigene panels), and mitochondrial genome analysis (1/34, 2.9%). In addition, 12 candidate genes in 14 patients (14/234, 6%) were identified by WES, of which 5 genes (*ALPL, CACNA1C, MED12, TCF4*, and *TCF20*) might partially explain clinical features and 7 (*CD99L2, TAF1, CLCN6, CYFIP1, GPT2, ATP2A2*, and *MYO18A*) were identified as relative risk genes.

### The Underlying Etiologies Based on the NISC Categories

Overall, 288 (53.2%) cases were identified to have underlying etiology: structural-acquired, 137 (25.3%); genetic, 70 (12.9%); genetic-structural, 39 (7.2%); structural-congenital, 27 (5.0%); metabolic, 13 (2.4%); and infections, 2 (0.4%). None of the cases showed immune etiology (Table 2).

**Table 2:** The specific causes of the group (*N* = 541).

**Etiologic categories (*n*)**	**Specific causes**	***N***
Structural-acquired (*n* = 137)		
	HIE with or without ICH/hypoglycemia	
	Perinatal insult	62
	Postnatal insult	8
	Intracranial infection	
	Bacterial meningitis (perinatal)	9 (1)
	Viral meningoencephalitis	8
	Brain injury secondary to neonatal hypoglycemia	18
	ICH	
	Perinatal insult	13
	Postnatal insult	3
	Encephalomalacia with other causes	
	Indefinite perinatal insult	2
	CVM	2
	Incontinentia pigmenti	1
	Unknown causes	8
	Neuroglioma	2
	Focal brain lesion of unknown cause	1
Genetic-structural (*n* = 39)		
	Tuberous sclerosis complex	
	*TSC1* variant	4
	*TSC2* variant	10
	NA	17
	Neurofibromatosis	
	*NF1* variant	2
	NA	2
	*NEDD4L* variant (heterotopia, pachygyria-lissencephaly)	1
	*DCX* variant (pachygyria-lissencephaly, agenesis of the corpus callosum)	1
	*NPRL3* variant (pachygyria-lissencephaly)	1
	17p13.3 microdeletion (pachygyria-lissencephaly, heterotopia)	1
Structural-congenital (*n* = 27)		
	Pachygyria-lissencephaly	12
	Focal cortical dysplasia	4
	Heterotopia	2
	Polymicrogyria	1
	Schizencephaly	1
	≥2 Malformations	
	Pachygyria-lissencephaly, heterotopia, schizencephaly, agenesis of the corpus callosum	1
	Heterotopia, focal cortical dysplasia, agenesis of the corpus callosum	1
	Heterotopia, agenesis of the corpus callosum, encephalomalacia with unknown cause (not epileptogenicity)	1
	Focal cortical dysplasia, schizencephaly	1
	Intracranial hemangioma	3
Genetic (*n* = 70)		
	*STXBP1* variant	12
	*CDKL5* variant	12
	*KCNQ2* variant	5
	*CLCN4* variant	3
	*IRF2BPL* variant	4
	*GNAO1* variant	2
	*SCN8A* variant	2
	*KCNB1* variant	2
	*SCN2A* variant	2
	*SCN10A* variant	1
	*CYFIP2* + *KMT2D* variant	1
	*MECP2* variant	1
	*DNM1* variant	1
	*ARX* variant	1
	*GRIN2B* variant	1
	*AARS* variant	1
	*NTRK2* variant	1
	*SPTAN1* variant	1
	*CACNA1A* variant	1
	*GNB1* variant	1
	*GABRE* variant	1
	*KMT2D* variant	1
	*UFC1* variant	1
	*SMARCA2* variant	1
	Xp22.13 microdeletion (harbors the exon 1 of the *CDKL5* gene)	1
	20q13.33 microdeletion (harbors the *EEF1A2, KCNQ2* genes)	1
	9q33.3-34.11 microdeletion (harbors the *STXBP1* gene)	1
	9p24.3-22.3 microdeletion	1
	5p12-11 microduplication (harbors the *HCN1* gene)	1
	3p25.3 microdeletion (harbors the *SETD5* gene)	1
	1p36.33 microdeletion (harbors the *GNB1* gene)	2
	1p36.33-32 microdeletion (harbors the *GNB1* gene)	1
	Xp22.11-21.3 microduplication (harbors the *ARX* gene)	1
	15q11.2 microduplication	1
Metabolic (*n* = 13)		
	Disorder of glycosylation	
	*SLC35A2* variant	2
	*ALG1* variant	1
	*ALG13* variant	1
	Metal metabolism	
	Menkes disease (*ATP7A* variant)	1
	Neurodegeneration with brain iron accumulation (*WDR45* variant)	3
	MMA (*MMACHC* variant)	1
	Pyridoxine-dependent epilepsy (*ALDH7A1* variant)	1
	SCAD deficiency (*ACADS* variant)	1
	Lysosomal storage diseases	
	*HEXA* variant	1
	Mitochondrial disorders (*MT-ND1* variant)	1
Infection (*n* = 2)		
	Intra-uterine infection	2
Unknown (*n* = 253)		
	Likely genetic	
	*CD99L2* variant	2
	*TAF1* variant	2
	*GPT2* variant	1
	*ATP2A2* variant	1
	*CYFIP1* variant	1
	*MYO18A* variant	1
	*CLCN6* variant	1
	*CACNA1C* variant	1
	*MED12* variant	1
	*TCF4* variant	1
	*TCF20* variant	1
	Likely metabolic (supporting evidence)	
	Pyridoxine-dependent epilepsy (resolved with B6 supplementation)	1
	GLUT-1 deficiency syndrome (hypoglycorrhachia)	1
	Leukoencephalopathy (lesions in brain MRI)	1
	Hypophosphatasia (*ALPL* variant)	1
	Others	236

*CVM, cerebral venous malformation; GLUT, glucose transporter; HIE, hypoxic-ischemic encephalopathy; ICH, intracranial hemorrhage; MMA, methylmalonic academia; MRI, magnetic resonance imaging; NA, not applicable; SCAD, short-chain Acyl-CoA dehydrogenase*.

Ninety-six (70.1%) of the 137 structural-acquired cases were caused by perinatal brain injuries, with hypoxic-ischemic encephalopathy (HIE) (51.1%, 70/137) and hypoglycemic brain injuries (13.1%, 18/137) being the most common causes. Of the 66 genetic/congenital-structural patients, 59 (89.4%) exhibited malformations of cortical development and the most common one was tuberous sclerosis complex (TSC) (31/59, 52.5%). The data of genetic tests were available in 20 of the 39 individuals with genetic-structural etiologies, among which *TSC2* (10), *TSC1* (4), and *NF1* (2) were the major associated genes.

Of the 70 genetic IS patients, 59 had monogenic variants and 11 chromosomal aberrations. The common genes were *STXBP1* (12), *CDKL5* (12), and *KCNQ2* (5). Seven of the 13 metabolic IS patients exhibited inborn metabolic errors in organic molecules, including metal metabolism (4/13, 30.7%), amino acid metabolism (1/13, 7.7%), vitamin B6 insufficiency (1/13, 7.7%), and fatty acid oxidation disorder (1/13, 7.7%). Besides, 4 (30.8%) cases had glycosylation disorders and 2 (15.4%) errors of metabolism in organelles. Ten genes (*WDR45, SLC35A2, ALG1, ALG13, ATP7A, HEXA, MMACHC, ALDH7A1, ACADS*, and *MT-ND1*) were implicated in metabolic IS.

### Secondary Findings

An overview of the available genetic data from 103 patients with genetic disorders (genetic-structural, genetic alone, and metabolic IS) revealed that monogenic variants accounted for 88.3% (91/103) and chromosomal aberrations for 11.6% (12/103) (Supplementary Tables S1, S2). Taking into account genes spanned by chromosomal aberrations, a total of 43 causative genes were identified in these 103 individuals (Supplementary Figure S2). *STXBP1* (13), *CDKL5* (13), *TSC2* (10), *KCNQ2* (6), *IRF2BPL* (4), and *TSC1* (4) were the most frequently implicated genes, which were responsible for 48.5% of the 103 patients with genetic disorders, while other causative genes were found in <3% respectively.

Seventeen (39.5%) of the identified 43 genes were DEE-related genes recorded in the OMIM database, whereas the remaining 26 (60.5%) genes were associated with other diseases. To facilitate an understanding of genetic pathogenesis, the mutated genes were divided into seven groups according to their functions (Supplementary Table S3), with genes encoding for ion transmembrane transport being the most prevalent (12/43, 27.9%). In terms of treatability, *TSC1*-, *TSC2*-, *SLC35A2*-, *ATP7A*-, *ALDH7A1*-, and *MMACHC*-related disorders had specific therapeutic regimens and accounted for 18.5% (19/103) of the cases with genetic disorders. Correspondingly, there were preferred drugs for patients with causal variants in *KCNQ2, SCN2A, SCN8A, CLCN4*, such as sodium channel blockers, accounting for 11.7% (12/103) of the 103 cases. In addition, therapies for *STXBP1*-, *CDKL5*-, *NF1*-, *MECP2*-, *DNM1*-, *GRIN2B*-, *HEXA*-related disorders were under investigation.

### Etiology and Distribution of IS

In comparison to females, males had a higher proportion of structural-acquired etiologies (*p* = 0.006) and a lower proportion of genetic (*p* < 0.001) and metabolic etiologies (*p* = 0.024). In contrast to term babies, preterm babies had a higher proportion of structural-acquired etiologies (*p* = 0.001). Patients with hypsarrhythmia had a significantly higher ratio of non-acquired structural etiologies (structural-congenital and genetic-structural causes) (*p* = 0.022) compared with those without hypsarrhythmia. Patients with pre-spasm seizures had significantly higher proportions of structural causes (*p* = 0.007), genetic causes (*p* < 0.001), and a lower proportion of unknown causes (*p* < 0.001) compared with cases without pre-spasm seizures. Early-onset IS group had a higher ratio of genetic causes, compared with classic onset IS (*p* < 0.001) and late-onset IS (*p* = 0.006). All other factors showed no significant differences. Supplementary Table S4 shows the etiological distribution of different IS groups.

**Figure 2:**
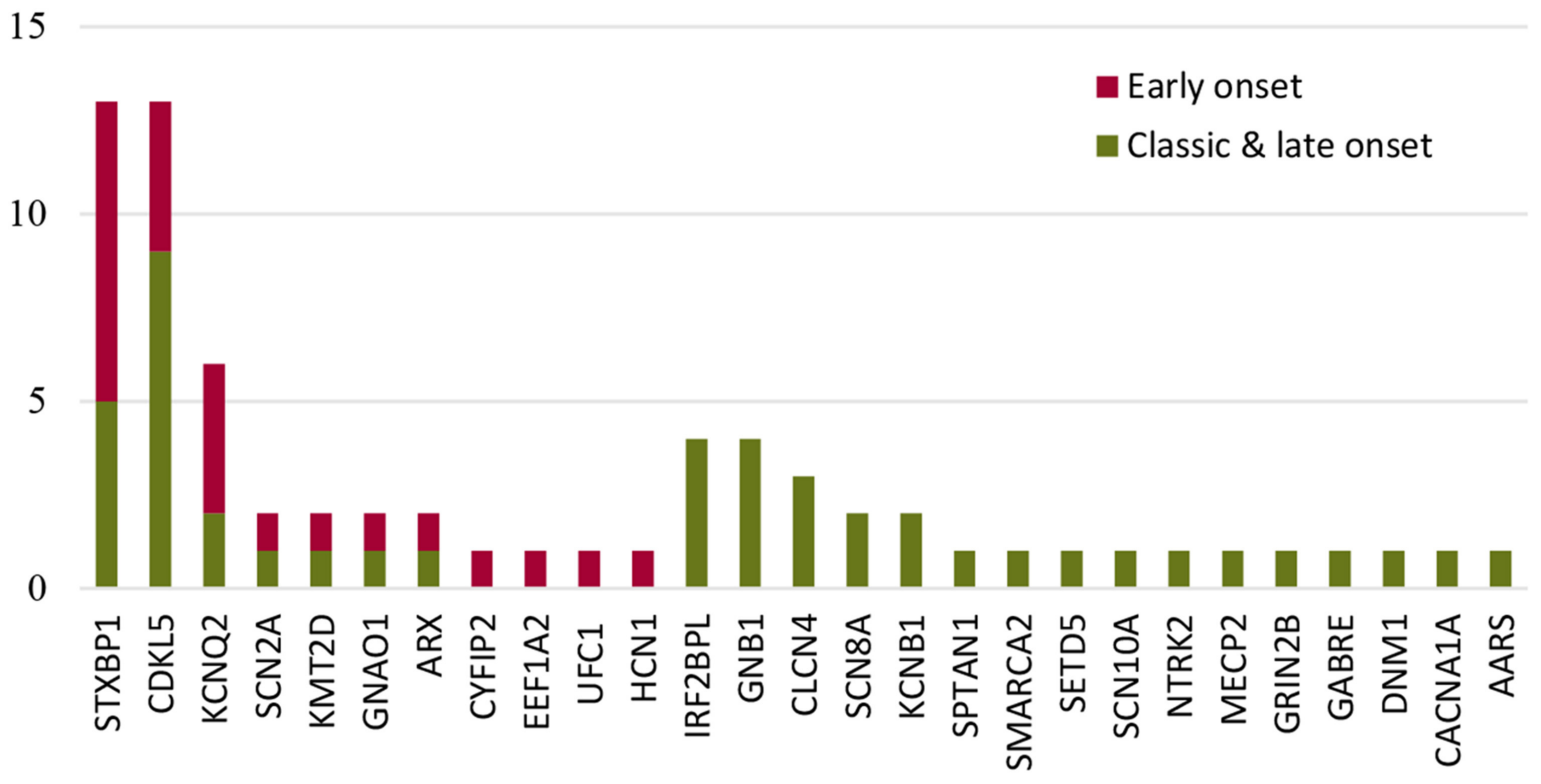
The distribution of the most common genes in the genetic IS group according to the ages of onset.

The most common causative gene in the early-onset genetic IS group was *STXBP1*, while *CDKL5* was the causative gene for classic and late-onset (Figure 2). For neonatal-onset IS (*n* = 13), the leading causes were structural-acquired followed by genetic. Variants in *KCNQ2, STXBP1*, and *SCN2A* were responsible for the 3 cases presenting with neonatal-onset spasms, respectively. The distribution of the genes in the genetic IS group was investigated as shown in Figure 2. For the 13 cases with *STXBP1* variants, eight cases had spasms onset within the third month of age, and the distribution showed a statistically significant difference when compared with other genes (*p* = 0.017). In contrast, nine of the 13 cases with *CDKL5* variants presented with spasms beyond the early-onset period, however, the distribution showed no statistically significant difference with other genes (*p* = 0.736).

## Discussion

To the best of our knowledge, this is the largest cohort study that summarizes the underlying etiologies of IS. The study found that 53.4% of the cases had an underlying etiology. Correspondingly, 64.4 and 58% of the cases in NISC and International Collaborative Infantile Spasms Study (ICISS) studies, respectively, had an identified cause (6, 8). In conjunction with NISC, the leading group was structural acquired followed by genetic IS, and in both studies, there was no immune cause. This study also highlighted the efficacy of brain MRI as a first-line investigation (yield, 37.5%). Furthermore, there are still some differences when comparing the listed cohorts, which might be characteristic of the Chinese population.

Neonatal hypoglycemic brain injury remains an important cause of epilepsy in developing nations, especially for West syndrome, although it has been excluded in studies done in developed nations. The advanced perinatal care and routinely monitored blood glucose in the Western world might be the explanation for the difference (11). The proportion of hypoglycemic brain injuries in structural-acquired IS in this study was much higher than in previous reports, ranking second only to HIE, indicating the importance of strengthening routine glucose monitoring and hypoglycemia management in perinatal care in China.

Down syndrome is one of the major causes of infantile spasms, contributing to around 10–20% of all known etiologies (6, 8, 12). Surprisingly, Down syndrome was not found in our sample. The disparity might be explained by the introduction of non-invasive prenatal screening for Down syndrome in China, as well as a decrease in the number of live births in infants with Down syndrome (13).

Chromosomal aberrations were only identified in a few of the patients while monogenic variants accounted for a vast majority of the genetic disorders. The most commonly implicated genes were *STXBP1, CDKL5, TSC2, KCNQ2, IRF2BPL*, and *TSC1*, which accounted for almost half of the patients with genetic disorders in our study. Except for the genes linked to TSC, the rest were highly consistent with the major genes highlighted in the group with early-onset epilepsies and/or early-onset epileptic encephalopathy with burst suppression (14–16). In retrospect, patients with spasms onset <2 months and those who had previously been diagnosed with Ohtahara syndrome were included in this study. As a result, differences in the major causative genes between studies should be attributed to the broader inclusion criteria used. Differs from the high-frequency genes, other causative genes (ARX, SCN2A, etc.) were only found in <3% of the cases with genetic disorders, respectively. It is evident that IS is highly heterogeneous in terms of genetic etiology. Moreover, the majority of genes involved in our group also showed phenotypic heterogeneity, as it occurs with other neurological disorders in OMIM.

The proportion of metabolic disorders was low in this study, as well as in the NISC study (8). Literature shows that the frequency of inborn errors of metabolism in infants with spasms varies widely (3–22%), depending on the number of patients enrolled and the extent of investigations investigations (4, 8, 17, 18). The fact, that majority of the cases in our cohort showed non-specific changes in routine metabolic screening and were discovered by genetic testing, reflects that metabolic etiologies are likely to be the overlooked contributors of IS, particularly in regions where genetic testing is not available and/or affordable. Genetic testing should, therefore, be considered for cases suspected to have metabolic IS.

As reported, WES explained 28% of cases with IS and also revealed 1–3 *de novo* variants with interesting candidate genes in 64% of the remaining cases (19). In the current study, about 6.0% of the cases that underwent WES revealed 12 candidate genes (*ALPL, CACNA1C, MED12, TCF4, TCF20, CD99L2, TAF1, CLCN6, CYFIP1, GPT2, ATP2A2*, and *MYO18A*) which need to be confirmed by reanalyzing the data and functional studies. *CYFIP2* was identified as the causative gene for DEE 65 in OMIM soon after we recognized and reported it as a causative gene for IS (20, 21). Thus, despite the customized multigene panels, WES had much higher diagnostic yields compared to other genetic tests in our study. WES should therefore be considered as the most suitable test when brain MRI fails to detect the underlying etiology. WES is cost-effective, can detect a wide range of genes, and the results can be re-analyzed with time.

The distribution of etiologies is influenced by different factors. Both males and preterm babies independently had higher proportions of structural-acquired etiologies. The male predominance in many acquired diseases such as viral/bacterial meningitis, other uncommon infections, and neonatal stroke (22–25) in children and the association between the low gestational age and high incidence of perinatal brain injury (26, 27) might explain the aforementioned observation in different genders and gestational ages.

Genetic causes play an important role (>20%) in initiating early-onset IS compared with other onset ages. The distribution of *STXBP1* was concentrated in early-onset IS cases (*P* = 0.017). Except for cases with acquired-structural abnormalities, genetic etiology was also a major contributor to neonatal IS and the involved genes were *STXBP1, KCNQ2*, and *SCN2A*. Knowledge of the common causative genes for early-onset IS and neonatal IS might guide clinicians in prescribing precise medication for cases lacking genetic results.

Cases without hypsarrhythmia had a higher ratio of non-acquired structural etiologies, and the reason for this is currently unknown. Patients with previous seizures had a lower proportion of unknown causes and higher proportions of structural-acquired and genetic causes. Consistent with our present study, cases with previous seizures have a high proportion of known etiology (5). The NISC study (2017) indicates that preexisting epilepsy reduces the likelihood of receiving standard therapy, including adrenocorticotropic hormone, prednisolone, or vigabatrin and is associated with low treatment response (7).

## Limitations

This study is the largest cohort study that attempts to investigate the etiology and distribution of IS. However, it is limited by the fact that it was retrospective thus, prone to bias. Besides, the sample size is limited to cases from 18 central-southern Chinese provinces thus, the findings cannot be generalized to other geographical locations.

## Conclusions

More than half of the IS cases in China had an underlying etiology. The most common cause was structural-acquired followed by genetic causes. HIE and hypoglycemic brain injuries were the major causes of structural-acquired IS. Down syndrome was absent in this cohort. Monogenic variants were very heterogeneous. The most commonly implicated genes were *STXBP1, CDKL5, TSC2, KCNQ2, IRF2BPL*, and *TSC1*. WES had a diagnostic yield of 26.9% and should be explored when brain MRI fails to find the underlying cause.

The etiological makeup differed in different scenarios. Male and preterm patients were more likely to have a structural-acquired etiology. Early-onset IS cases had a higher ratio of genetic causes. There was a significant proportion of known etiology in those with prior seizures. Cases without hypsarrhythmia had higher ratios of non-acquired structural etiologies compared with those with hypsarrhythmia.

## Data Availability Statement

The original contributions presented in the study are included in the article/Supplementary Material, further inquiries can be directed to the corresponding author/s.

## Ethics Statement

The studies involving human participants were reviewed and approved by the Institutional Ethics Committee of Xiangya Hospital, Central South University. Written informed consent to participate in this study was provided by the participants' legal guardian/next of kin.

## Author Contributions

PP collected, analyzed the data, and drafted the initial manuscript. MK drafted and revised the final manuscript. LM and ZP collected data and carried out the initial analyses. FH, CZ, CC, NP, and FY coordinated and supervised data collection. JP and ZP conceptualized and designed the study, coordinated and supervised data collection, and critically reviewed the manuscript for important intellectual content. All authors reviewed the manuscript and approved the submitted version (and any substantially modified version that involves the author's contribution to the study) and have agreed to be personally accountable for the author's contributions and to ensure that questions related to the accuracy or integrity of any part of the work, even ones in which the author was not personally involved, are appropriately investigated, resolved, and the resolution documented in the literature.

## Funding

This study was funded by the National Natural Science Foundation of China (grant numbers 81771409 and 82071462), and the Hunan Province Key Technology Support Program (2015SK2019).

## Conflict of Interest

The authors declare that the research was conducted in the absence of any commercial or financial relationships that could be construed as a potential conflict of interest.

## Publisher's Note

All claims expressed in this article are solely those of the authors and do not necessarily represent those of their affiliated organizations, or those of the publisher, the editors and the reviewers. Any product that may be evaluated in this article, or claim that may be made by its manufacturer, is not guaranteed or endorsed by the publisher.
