# Supplementary material for: Etiologic Classification of 541 Infantile Spasms Cases: A Cohort Study
Source: Front Pediatr. 2022 Mar 7;10:774828. doi: 10.3389/fped.2022.774828 (PMC8940518; doi:10.3389/fped.2022.774828)
Supplement: Supplementary file 1 [file Data_Sheet_1.docx]

| **Table S1. A list of gene variants identified in the group with identified causes.** | | | | | | | | | | |
| --- | --- | --- | --- | --- | --- | --- | --- | --- | --- | --- |
| **Patient ID** | **Sex** | **Gene** | **Transcript** | **Gene location** | **cDNA change** | **Protein change** | **Gene region** | **Zygosity** | **Inheritance** | **ACMG classification** |
| 54 | F | *STXBP1* | NM_003165 | chr9:130416034-130416036 | c.128_130del | p.S43del | exon3 | het | De novo | LP |
| 126 | F | *STXBP1* | NM_003165 | chr9:130438199-130438201 | c.1227_1229del | p.L410del | exon14 | het | De novo | LP |
| 197 | M | *STXBP1* | NM_003165 | chr9:130438188 | c.1216C>T | p.R406C | exon14 | het | De novo | P |
| 240 | F | *STXBP1* | NM_003165 | chr9:130428514 | c.733C>A | p.H245N | exon9 | het | De novo | P |
| 281 | M | *STXBP1* | NM_003165 | chr9:130442467-130442479 | c.1493_1505del | p.H498Pfs*44 | exon17 | het | De novo | LP |
| 304 | F | *STXBP1* | NM_003165 | chr9:130434396 | c.1029+1G>T | splicing | exon12 | het | De novo | P |
| 369 | F | *STXBP1* | NM_003165 | chr9:130434297 | c.664-1G>- | splicing | exon9 | het | De novo | P |
| 416 | F | *STXBP1* | NM_003165 | chr9:130438188 | c.1216C>T | p.R406C | exon14 | het | De novo | P |
| 420 | M | *STXBP1* | NM_003165 | chr9:130440807 | c.1457T>G | p.M486R | exon16 | het | De novo | LP |
| 109 | F | *STXBP1* | NM_003165 | chr9:130416028 | c.122T>G | p.L41R | exon3 | het | De novo | LP |
| 153 | M | *STXBP1* | NM_003165 | chr9:130428503 | c.722C>T | p.S241F | exon9 | het | De novo | LP |
| 540 | M | *STXBP1* | NM_003165 | chr9:130428484 | c.703C>T | p.R235* | exon9 | het | De novo | P |
| 25 | F | *CDKL5* | NM_003159 | chrX:18646629-18646630 | c.2635_2636del | p.L879Efs | exon18 | het | De novo | P |
| 247 | F | *CDKL5* | NM_003159 | chrX:18598086 | c.401G>C | p.R134P | exon6 | het | De novo | P |
| 300 | M | *CDKL5* | NM_003159 | chrX:18593610 | c.282dup | p.N95Kfs*16 | exon5 | hem | De novo | P |
| 383 | F | *CDKL5* | - | - | exon4-9 del | - | exon4-9 | het | De novo | P |
| 461 | F | *CDKL5* | NM_003159 | chrX:18622155 | c.1111del | p.A372Lfs*121 | exon12 | het | De novo | P |
| 479 | F | *CDKL5* | NM_003159 | chrX:18525274 | c.58G>C | p.G20R | exon2 | het | De novo | P |
| 487 | F | *CDKL5* | NM_003159 | chrX:18622950 | c.1906del | p.A636Qfs*22 | exon13 | het | De novo | P |
| 128 | M | *CDKL5* | NM_003159 | chrX:18606139 | c.620G>A | p.G207E | exon9 | hem | De novo | P |
| **Patient ID** | **Sex** | **Gene** | **Transcript** | **Gene location** | **cDNA change** | **Protein change** | **Gene region** | **Zygosity** | **Inheritance** | **ACMG classification** |
| 412 | F | *CDKL5* | NM_003159 | chrX:18528975 | c.99+1G>A | splicing | exon3 | het | De novo | P |
| 450 | F | *CDKL5* | NM_003159 | chrX:18622563 | c.1519C>T | p.Q507* | exon12 | het | De novo | P |
| 509 | M | *CDKL5* | NM_003159 | chrX:18622374 | c.1330del | p.R444Afs*49 | exon12 | hem | De novo | P |
| 516 | F | *CDKL5* | NM_003159 | chrX:18631309 | c.2190dup | p.N731* | exon15 | het | De novo | P |
| 24 | F | *KCNQ2* | NM_172107 | chr20:62044927 | c.1639C>T | p.R547W | exon15 | het | De novo | P |
| 42 | F | *KCNQ2* | NM_172107 | chr20:62076109 | c.593G>A | p.R198Q | exon4 | het | De novo | P |
| 453 | F | *KCNQ2* | NM_172107 | chr20:62078146 | c.341C>T | p.T114I | exon2 | het | De novo | P |
| 175 | F | *KCNQ2* | NM_172107 | chr20:62044854 | c.1712A>C | p.Y571S | exon15 | het | De novo | LP |
| 270 | M | *KCNQ2* | NM_172107 | chr20:62076133 | c.569A>G | p.N190S | exon4 | het | De novo | LP |
| 46 | M | *CLCN4* | NM_001256944 | chrX:10176604 | c.1081G>A | p.V361I | exon7 | hem | Maternally inherited | VUS |
| 52 | M | *CLCN4* | NM_001256944 | chrX:10181739 | c.1313C>A | p.T438K | exon9 | hem | De novo | LP |
| 84 | M | *CLCN4* | NM_001256944 | chrX:10182017 | c.1591C>T | p.L531F | exon9 | hem | Maternally inherited | LP |
| 12 | F | *IRF2BPL* | NM_024496 | chr14:77492741-77492743 | c.1393_1395del | p.K465del | exon1 | het | De novo | LP |
| 251 | F | *IRF2BPL* | NM_024496 | chr14:77491999 | c.2137del | p.L713Sfs*54 | exon1 | het | De novo | LP |
| 385 | F | *IRF2BPL* | NM_024496 | chr14:77493003 | c.1133C>A | p.T378K | exon1 | het | De novo | LP |
| 415 | F | *IRF2BPL* | NM_024496 | chr14:77493904 | c.232del | p.V78Sfs*74 | exon1 | het | De novo | P |
| 152 | F | *GNAO1* | NM_138736 | chr16:56226522 | c.155A>G | p.Q52R | exon2 | het | De novo | P |
| 398 | F | *GNAO1* | NM_138736 | chr16:56370656 | c.607G>A | p.G203R | exon6 | het | De novo | P |
| 82 | M | *SCN8A* | NM_001177984 | chr12:52099294 | c.1228G>A | p.V410M | exon10 | het | De novo | LP |
| **Patient ID** | **Sex** | **Gene** | **Transcript** | **Gene location** | **cDNA change** | **Protein change** | **Gene region** | **Zygosity** | **Inheritance** | **ACMG classification** |
| 184 | M | *SCN8A* | NM_001177984 | chr12:52082596 | c.669G>C | p.R223S | exon6 | het | De novo | LP |
| 488 | M | *KCNB1* | NM_004975 | chr20:47990967 | c.1130C>T | p.T377I | exon2 | het | De novo | LP |
| 269 | F | *KCNB1* | NM_004975 | chr20:47991526 | c.571del | p.A192Pfs*2 | exon2 | het | De novo | LP |
| 63 | M | *SCN2A* | NM_001040143 | chr2:166170515 | c.1280C>A | p.A427D | exon9 | het | De novo | VUS |
| 498 | F | *SCN2A* | NM_021007 | chr2:166166942 | c.807G>T | p.L269F | exon7 | het | De novo | LP |
| 23 | F | *SCN10A* | NM_006514 | chr3:38739687 | c.5024C>T | p.P1675L | exon27 | het | Paternally  inherited | VUS |
|  |  |  |  | chr3:38781125 | c.2161C>T | p.P721S | exon14 | het | Maternally inherited | VUS |
| 245 | F | *CYFIP2* | NM_001037333 | chr5:156721843 | c.259C>T | p.R87C | exon4 | het | De novo | LP |
|  |  | *KMT2D* | NM_003482 | chr12:49427485 | c.11003C>T | p.P3668L | exon39 | het | De novo | LP |
| 127 | F | *MECP2* | - | - | exon1 dup | - | exon1 | het | Unknown | P |
|  |  |  | - | - | exon2-3 del | - | exon2-3 | het | Unknown | P |
| 327 | M | *DNM1* | NM_001005336 | chr9:130981385 | c.443A>G | p.Q148R | exon4 | het | De novo | P |
| 491 | M | *ARX* | NM_139058 | chrX:25022876 | c.1600G>C | p.A534P | exon5 | hem | Maternally inherited | P |
| 489 | M | *GRIN2B* | NM_000834 | chr12:13761625 | c.1922T>C | p.I641T | exon10 | het | De novo | LP |
| 490 | M | *AARS* | NM_001605 | chr16:70310958 | c.244C>T | p.H82Y | exon3 | het | Maternally inherited | VUS |
|  |  |  |  | chr16:70303522 | c.961G>C | p.G321R | exon7 | het | Paternally  inherited | VUS |
| 28 | M | *NTRK2* | NM_006180 | chr9:87366905 | c.1301A>G | p.Y434C | exon14 | het | De novo | LP |
| **Patient ID** | **Sex** | **Gene** | **Transcript** | **Gene location** | **cDNA change** | **Protein change** | **Gene region** | **Zygosity** | **Inheritance** | **ACMG classification** |
| 68 | F | *SPTAN1* | NM_001130438 | chr9:131389713-131389715 | c.6625_6627del | p.D2209del | exon50 | het | De novo | LP |
| 139 | M | *CACNA1A* | NM_001127222 | chr19:13319752-13319763 | c.6587_6598del | p.2196_2199del | exon46 | het | Maternally inherited | VUS |
| 282 | M | *GNB1* | NM_001282538 | chr1:1720572 | c.536C>T | p.S179F | exon8 | het | De novo | LP |
| 408 | M | *GABRE* | NM_004961 | chrX:151123382 | c.1312C>T | p.R438C | exon9 | hem | Maternally inherited | VUS |
| 337 | F | *KMT2D* | NM_003482 | chr12:49442971 | c.3937C>T | p.R1313W | exon12 | het | De novo | LP |
| 521 | M | *UFC1* | NM_016406 | chr1:161126779 | c.163C>T | p.R55* | exon2 | het | Maternally inherited | LP |
|  |  |  |  | chr1:161123902 | c.115C>T | p.L39F | exon1 | het | Paternally  inherited | VUS |
| 420 | M | *SMARCA2* | NM_003070 | chr9:2086853 | c.2551G>A | p.D851N | exon18 | het | De novo | LP |
| 5 | M | *TSC1* | NM_000368 | chr9:135781074-135781077 | c.1888_1891del | p.K630Qfs*22 | exon15 | het | De novo | P |
| 34 | M | *TSC1* | *-* | - | exon5 dup | - | exon5 | het | De novo | P |
|  |  |  | - | - | exon11 del | - | exon11 | het | De novo | P |
| 187 | F | *TSC1* | NM_000368 | chr9:135797340 | c.529C>T | p.L177F | exon7 | het | De novo | LP |
| 463 | F | *TSC1* | NM_000368 | chr9:135781440 | c.1525C>T | p.R509* | exon15 | het | De novo | P |
| 8 | M | *TSC2** | NM_000548 | chr16:2130220 | c.3452G>A | p.G1151D | exon29 | het | Maternally inherited | P |
| 83 | F | *TSC2* | NM_000548 | chr16:2103342 | c.226-1G>T | splicing | exon4 | het | De novo | P |
|  |  | *TSC2* | NM_000548 | chr16:2103343 | c.226C>T | p.H76Y | exon4 | het | De novo | VUS |
| 124 | M | *TSC2* | NM_000548 | chr16:2130180 | c.3412C>T | p.R1138* | exon30 | het | De novo | P |
| **Patient ID** | **Sex** | **Gene** | **Transcript** | **Gene location** | **cDNA change** | **Protein change** | **Gene region** | **Zygosity** | **Inheritance** | **ACMG classification** |
| 215 | F | *TSC2* | NM_000548 | chr16:2106693 | c.697dup | p.A233Gfs*2 | exon8 | het | De novo | P |
| 347 | M | *TSC2* | NM_000548 | chr16:2130220 | c.3452del | p.G1151Afs*40 | exon30 | het | De novo | P |
| 428 | F | *TSC2* | NM_000548 | chr16:2112001 | c.1249C>T | p.Q417* | exon12 | het | De novo | P |
| 446 | M | *TSC2* | NM_000548 | chr16:2112576 | c.1336C>T | p.Q446* | exon13 | het | De novo | P |
| 504 | F | *TSC2* | NM_000548 | chr16:2130210 | c.3442C>T | p.Q1148* | exon30 | het | De novo | P |
| 519 | M | *TSC2* | *-* | - | exon16-26 del | - | exon16-26 | het | De novo | P |
| 56 | M | *NF1* | NM_000267 | chr17:29550508 | c.1768del | p.M590Cfs*15 | exon16 | het | De novo | P |
| 537 | F | *NF1* | *-* | - | exon13 del | - | exon13 | het | Unknown | P |
| 433 | M | *NEDD4L* | NM_015277 | chr18:56057899 | c.2617G>A | p.E873K | exon28 | het | De novo | P |
| 443 | M | *DCX* | NM_178153 | chrX:110653394 | c.233G>A | p.R78H | exon2 | hem | Maternally inherited | P |
| 456 | M | *NPRL3* | NM_001077350 | chr16:188264 | c.3G>A | p.? | exon2 | het | Paternally  inherited | LP |
| 393 | M | *SLC35A2* | NM_001042498 | chrX:48762728 | c.458C>A | p.A153E | exon4 | het | De novo | VUS |
| 451 | F | *SLC35A2* | NM_001042498 | chrX:48762493 | c.692dup | p.W232Vfs*23 | exon4 | het | De novo | P |
| 55 | F | *ALG1* | NM_019109 | chr16:5129063 | c.863-2A>G | splicing | exon8 | het | Paternally  inherited | P |
|  |  |  |  | chr16:5133708-5133710 | c.1213_1215del | p.E405del | exon12 | het | Maternally inherited | LP |
| 26 | F | *ALG13* | NM_001099922 | chrX:110928268 | c.320A>G | p.N107S | exon3 | het | De novo | LP |
| 163 | M | *ATP7A* | NM_000052 | chrX:77244218 | c.601C>T | p.R201* | exon3 | hem | Maternally inherited | P |
| **Patient ID** | **Sex** | **Gene** | **Transcript** | **Gene location** | **cDNA change** | **Protein change** | **Gene region** | **Zygosity** | **Inheritance** | **ACMG classification** |
| 47 | F | *WDR45* | NM_007075 | chrX:48935557 | c.69C>A | p.C23* | exon4 | het | Maternally inherited | LP |
| 357 | F | *WDR45* | NM_007075 | chrX:48933232 | c.700C>T | p.R234* | exon9 | het | De novo | P |
| 417 | F | *WDR45* | NM_007075 | chrX:48932540-48932541 | c.1007_1008del | p.Y336Cfs*4 | exon12 | het | Unknown | P |
| 101 | M | *HEXA* | NM_000520 | chr15:72637878 | c.1435G>A | p.A479T | exon13 | hom | Parentally  inherited | VUS |
| 465 | F | *MMACHC* | NM_015506 | chr1:45974647 | c.609G>A | p.W203* | exon4 | hom | Parentally  inherited | P |
| 423 | F | *ALDH7A1* | NM_001182 | chr5:125912858 | c.563T>C | p.V188A | exon6 | het | Paternally  inherited | VUS |
|  |  |  |  | - | exon1 del | - | exon1 | het | Unknown | P |
| 231 | M | *ACADS* | *-* | - | IVS5+1G>A | - | - | hom | Unknown | LP |
| 510 | M | *MT-ND1* | NC_012920 | chrM: 3761 | m.3761C>A | p.S152* | ND1 | 81.4% | De novo | LP |

**Abbreviations**: chr, chromosome; del, deletion; dup, duplication; F, female; hem, hemizygous; het, heterozygous; hom, homozygous; LP, likely pathogenic; M, male; ND, NADH dehydrogenase; P, pathogenic; VUS, uncertain significance variants.

**Table S2. Summary of the established CNVs causing infantile spasms in our study cohort.**

| **Patient ID** | **Sex** | **Chr location** | **Starting point** | **Stopping point** | **Size, Mb** | **Type of the aberration** | **Inheritance** | **ACMG classification** |
| --- | --- | --- | --- | --- | --- | --- | --- | --- |
| 174 | F | 1p33.33 | 820001 | 2240000 | 1.4 | del | De novo | LP |
| 184 | M | 5p12-p11 | 44190962 | 46400935 | 2.2 | dup | Unknown | P |
| 351 | F | 9p24.3-p22.3 | 46587 | 14547633 | 14.5 | del | De novo | P |
| 399 | M | 9q33.3-q34.11 | 129855337 | 130456157 | 0.6 | del | De novo | P |
| 458 | F | 20q13.33 | 61517110 | 62325455 | 0.81 | del | De novo | P |
| 459 | F | Xp22.13 | 18239218 | 18507575 | 0.26 | del | De novo | P |
| 469 | F | 4p16.3-p15.31 | 75001 | 20599643 | 20.52 | dup | Unknown | P |
| 487 | F | 17p13.3 | 1855382 | 2972634 | 1.12 | del | De novo | P |
| 454 | F | 3p25.3 | - | - | 0.18 | del | De novo | LP |
| 143 | F | Xp22.11-p21.3 | 24443147 | 25095204 | 0.6 | dup | Unknown | LP |
| 284 | M | 15q11.2 | 21974835 | 23226254 | 1.2 | dup | Maternally inherited | LP |
| 541 | F | 1p36.32-p36.33 | 719608 | 2369515 | 1.61 | del | De novo | P |
| **Abbreviations:** chr, chromosome; CNV, copy number variation; del, deletion; dup, duplication; LP, likely pathogenic; P, pathogenic. | | | | | | | | |

| **Gene functions** | **Number of genes (N)** | **Gene name** |
| --- | --- | --- |
| Ion transmembrane transport | 12 | *KCNQ2, SCN2A, SCN8A, GABRE, HCN1, CLCN4, SCN10A, CACNA1A, NEDD4L, KCNB1, ATP7A, GRIN2B* |
| Cell growth and proliferation regulation | 9 | *NF1, TSC1, TSC2, AARS, NPRL3, NTRK2, DCX, SETD5, WDR45* |
| Cell metabolism | 8 | *ALDH7A1, SLC35A2, ACADS, HEXA, ALG1, ALG13,* *MMACHC, MT-ND1* |
| Transcriptional regulation | 6 | *ARX, MECP2, EEF1A2, KMT2D, SMARCA2, IRF2BPL* |
| Protein kinases regulation | 4 | *CDKL5, GNAO1, GNB1,UFC1* |
| Synaptic transmission | 3 | *DNM1, STXBP1, CYFIP2* |
| Cell-in-cell interaction | 1 | *SPTAN1* |

**Table S3. The categories of the genes according to their function.**

**Table S4. The distribution of etiologies in different IS subgroups.**

|  | Structural-acquired | Infection | Structural-congenital | Genetic-structural | Genetic | Metabolic | Unknown | Total |
| --- | --- | --- | --- | --- | --- | --- | --- | --- |
| Female | 40  (19.0%) | 0 | 12  (5.7%) | 19  (9.0%) | 42  (19.9%) | 9  (4.3%) | 89  (42.2%) | 211 |
| Male | 97  (29.4%) | 2  (0.6%) | 15  (4.5%) | 20  (6.1%) | 28  (8.5%) | 4  (1.2%) | 164  (49.7%) | 330 |
|  |  |  |  |  |  |  |  |  |
| Term birth | 115  (23.4%) | 1  (0.2%) | 25  (5.1%) | 39  (7.9%) | 65  (13.2%) | 11  (2.2%) | 235  (47.9%) | 491 |
| Preterm birth | 22  (44.0%) | 1  (2.0%) | 2  (4.0%) | 0 | 5  (10.0%) | 2  (4.0%) | 18  (36.0%) | 50 |
|  |  |  |  |  |  |  |  |  |
| Hypsarrhythmia on EEG | 121  (25.9%) | 2  (0.4%) | 20  (4.3%) | 31  (6.6%) | 63  (13.5%) | 13  (2.8%) | 217  (46.5%) | 467 |
| No hypsarrhythmia on EEG | 16  (21.6%) | 0 | 7  (9.5%) | 8  (10.8%) | 7  (9.5%) | 0 | 36  (48.6%) | 74 |
|  |  |  |  |  |  |  |  |  |
| Pre-spasm seizure | 49  (36.8%) | 1  (0.8%) | 8  (6.0%) | 6  (4.5%) | 32  (24.1%) | 3  (2.3%) | 34  (25.6%) | 133 |
| No pre-spasm seizure | 88  (21.6%) | 1  (0.2%) | 19  (4.7%) | 33  (8.1%) | 38  (9.3%) | 10  (2.5%) | 219  (53.7%) | 408 |
|  |  |  |  |  |  |  |  |  |
| Early-onset | 20  (23.3%) | 1  (1.2%) | 6  (7.0%) | 5  (5.8%) | 22  (25.6%) | 1  (1.2%) | 31  (36.0%) | 86 |
| Classic-onset | 101  (25.7%) | 1  (0.3%) | 18  (4.6%) | 32  (8.1%) | 43  (10.9%) | 9  (2.3%) | 189  (48.1%) | 393 |
| Late-onset | 16  (25.8%) | 0 | 3  (4.8%) | 2  (3.2%) | 5  (8.1%) | 3  (4.8%) | 33  (53.2%) | 62 |


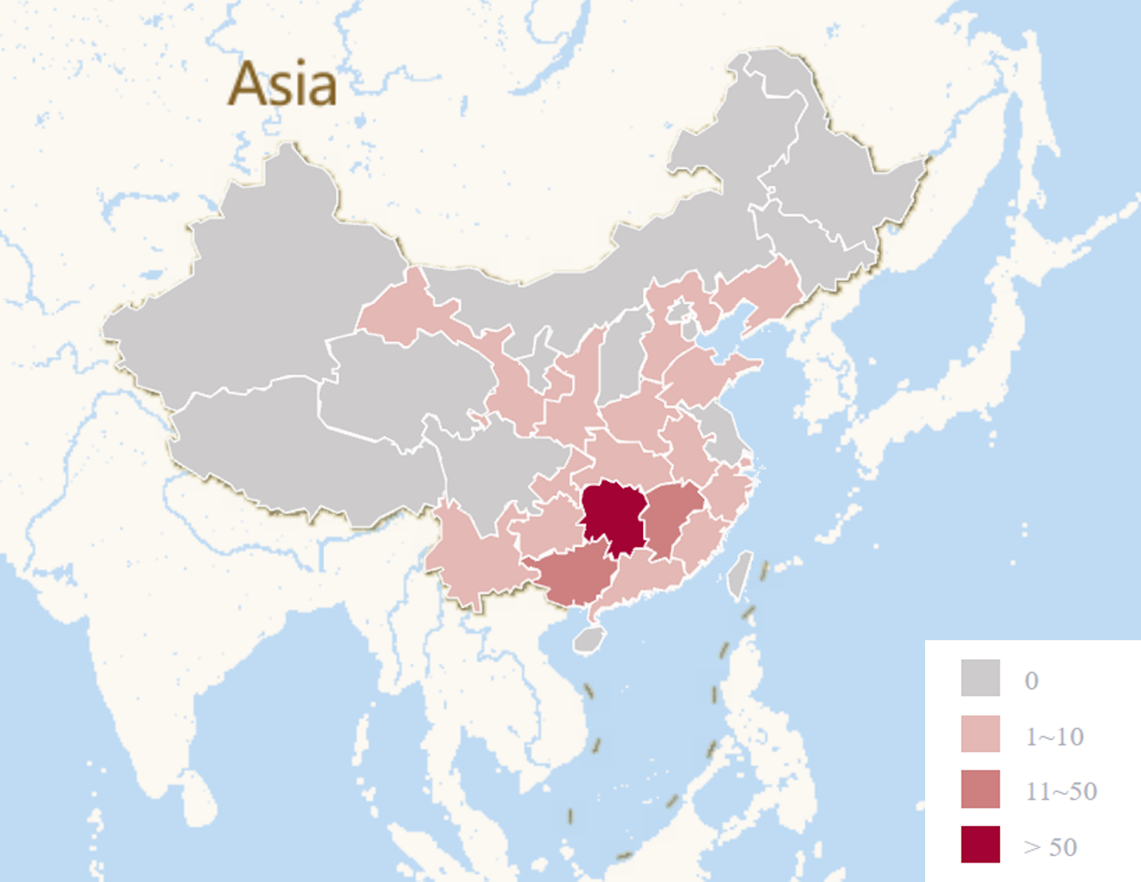


**Figure S1.The geographical distribution of the cohort.**

The enrolled 541 ISs patients came from 18 provinces, mainly concentrated in the central southern part of China.


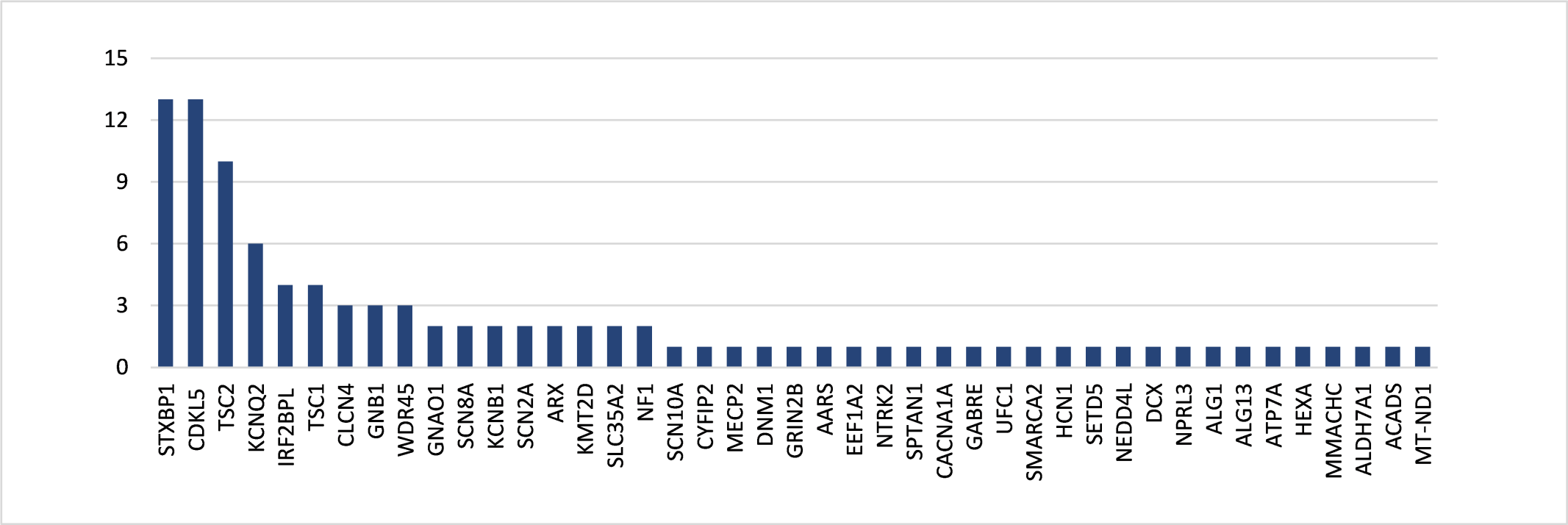


**Figure S2. The identified causative genes.**
